# Updates on Prevalence and Trend Status of Visceral Leishmaniasis at Two Health Facilities in Amhara Regional State, Northwest Ethiopia: A Retrospective Study

**DOI:** 10.1155/2022/3603892

**Published:** 2022-04-15

**Authors:** Mulat Yimer, Endalkachew Nibret, Gizachew Yismaw

**Affiliations:** ^1^Department of Medical Laboratory Sciences, School of Health Sciences, College of Medicine and Health Sciences, Bahir Dar University, Bahir Dar, Ethiopia; ^2^Department of Biology, College of Sciences, Bahir Dar University, Bahir Dar, Ethiopia; ^3^Amhara Public Health Institute, Bahir Dar, Ethiopia

## Abstract

Ethiopia is one of the countries accounted for over 90% of annual visceral leishmaniasis incidence. Despite this, yet there are no active and passive surveillance activities in the Amhara Region that will give up-to-date information about the disease status at the health facility levels. Therefore, this study aimed to report up-to-date information about visceral leishmaniasis and its trend status at two health facilities and the surrounding areas. A retrospective study from October 2017 to May 2021 was conducted by reviewing patient records at Metema and Addis Zemen Hospitals. Data on Sex, age, occupation, residence, month, year, and rK39 test results were collected using a questionnaire and were analyzed using Statistical Package for Social Sciences (SPSS) version 20. The chi-square test was used to see the association between variables. p < 0.05 was considered as statistically significant. Of the 2,703 visceral leishmaniasis suspected cases diagnosed with the rK39 test, 877 (32.4%) were confirmed (positive) cases. Monthly and yearly trends depicted that the largest number of suspected cases was reported in October and 2018, respectively. Daily laborers were the most affected individuals in Metema areas.

## 1. Introduction

Leishmaniases are groups of diseases caused by infection with protozoan parasites of the genus *Leishmania* [1] Although leishmaniases have been studied for over a century, the fight against them remains a hot topic [2]. It is transmitted by the bite of the female *Phlebotomus* species in the Old World and *Lutzomyia* species in the New World [3].

Depending on the infecting *Leishmania* species and/or the immune status of the host and some other factors, some are localized at the skin and resulted in cutaneous forms. Others infect visceral organs and cause visceral forms [4]. As a result, clinical manifestations of leishmaniases can be broadly grouped into two as visceral leishmaniasis (VL), caused by *L. donovani* complex (*L. donovani, L. infantum,* and/or *L. chagasi*), and cutaneous leishmaniasis (CL), caused by *L. major, L. tropica, L. aethiopica, L. braziliensis,* and *L. mexicana* species complex [5, 6].

Globally, leishmaniasis is prevalent in 98 countries and an estimated 350 million people are at risk of infection. The prevalence of the disease is 12 million with 0.2–0.4 million cases of VL and 0.7–1.2 million cases of CL [7, 8]. Recent reports depicted that in 2020, more than 90% of new VL cases reported to the World Health Organization (WHO) occurred in 10 countries: Brazil, China, Ethiopia, Eritrea, India, Kenya, Somalia, South Sudan, Sudan, and Yemen [9] (WHO leishmaniasis factsheet January 2022: https://www.who.int/news-room/fact-sheets/detail/leishmaniasis). In addition, East Africa is currently the most affected region in the world, accounting for 45% of visceral leishmaniasis cases reported to the WHO globally in 2018 [10].

Visceral leishmaniasis, also known as kala-azar, is one of the most neglected tropical diseases in the world and an estimated 50,000 to 90,000 new cases of VL annually occur worldwide, with only between 25 and 45% reported to the WHO (WHO leishmaniasis factsheet January 2022: https://www.who.int/news-room/fact-sheets/detail/leishmaniasis). Hence, this did not reflect the true burden of the disease due to under-reported and lack of active surveillance [11]. As the result, it inflicts an immense toll on the developing world and impedes economic development, with an estimated annual loss of 2.3 million disability-adjusted life years (DALYs) [11].

In Ethiopia, VL is a growing health problem that is continually spreading to new foci [12]. Visceral leishmaniasis is endemic in 40 districts of the country with annual cases of 2,500 to 4,000, and 3.2 million people are at risk. It has been known in southwest Ethiopia since 1942 [13] and has yet been remained endemic [11]. In northwest Ethiopia, it was first reported in Humera Hospital [14] and has yet been remained endemic [10]. In Ethiopia, VL is transmitted by sand fly vectors of *Phlebotomus martini* and *P. celiae* in the southwest and southern parts, whereas in the northwest and northern parts of the country, it is transmitted by *P. orientalis* [15, 16].

Several immunological diagnostic tools for the diagnosis of VL have been recently developed [17] and replaced parasitological methods for the diagnosis of VL, and they are sensitive [18]. Although several methods have been employed for serodiagnosis of VL, a 39-amino-acid-repeat recombinant leishmanial antigen from *Leishmania chagasi* (rK39) remains still the most feasible and commonly used method in endemic countries [19].

Over the last few years, Ethiopia has performed a lot of activities in the prevention and control of leishmaniasis (especially visceral leishmaniasis). Some of those activities have focused on targeting sand fly vectors, the use of long-lasting insecticide-treated nets, and the early diagnosis and treatment of cases [20]. Above all, up-to-date information about the disease was not reported timely due to the lack of active and passive surveillance activities, at the community and health facility levels, respectively.

Although VL is found in most parts of the Amhara Region (unpublished data), there have not been continuously published reports from health facilities in areas where the disease is prevalent. Some years back, however, the statuses of VL from Addis Zemen Health Centre in 2014 [21] and Metema Hospital in 2016 [22] were reported. Since then, the status of VL at these two health facilities has not been reported and its status has not yet been known. Moreover, this study will have some contribution to the control and elimination of VL that will be launched in East Africa (WHO, 2020). Furthermore, it will also contribute to the control of VL in Ethiopia. Finally, it will give baseline data for the active and passive surveillance activities in the Amhara Region. Therefore, the present retrospective study depicted updated information on the prevalence and trend status of VL at each health facility for designing effective intervention methods in and around the study areas.

## 2. Materials and Methods

### 2.1. Study Design, Area, and Period

A four-year and seven-month (October 2017- May 2021) retrospective study was conducted by reviewing laboratory registration books from Metema and Addis Zemen Hospitals.

Metema is one of the districts in the Amhara Region of Ethiopia, it was previously part of the North Gondar zone, and now it is part of the West Gondar zone. This district has a total population of 110,252. Of whom, 58,748 and 51,504 are men and women, respectively [23]. This area has been endemic to VL since 1942 [13]. The district has an altitude ranging from 550 to 1,600 m above sea level and a mean annual temperature ranging from 22 °C to 28 °C. The annual rainfall ranges from 400 to 600 mm (person. comm). The natural vegetation of this district is predominantly acacia trees with grasses grown under them, and most of the soil is clay soil. A lot of small- and large-scale private and government-owned farms are in the district. Because of these opportunities, many daily laborers travel each year from June to December from the highland, VL nonendemic areas to this lowland area, which is endemic to VL for weeding and harvesting of sesame, cotton, and corn [20]. Metema Hospital serves as a referral hospital for 14 health centers and daily laborers who come to the area for the weeding and harvesting seasons.

**Figure 1:**
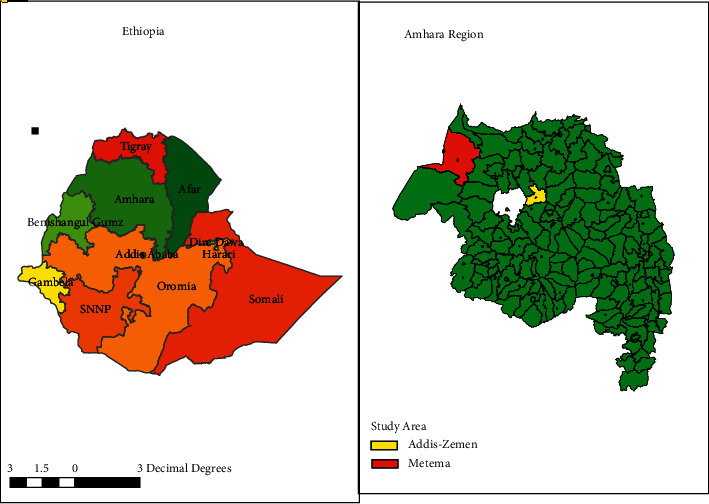
Map depicting the study areas.

Addis Zemen is found in the South Gondar administration zone in the Amhara Region of northwestern Ethiopia and has been endemic for VL since 2005 [24]. Addis Zemen is the capital city of the Libokemkem district. This district has a total population of 198,435. Of whom, 100,987 and 97,448 are men and women, respectively [23]. Teff, beans, cotton, and maize are the main agricultural crops. Most of the areas are water logged, by floods from the surrounding hills, during the rainy season (June to October), but the land dries up during the dry season (November to May), resulting in deep cracks in the soil surface. It has an altitude of 2,000 m above sea level. The mean annual temperature ranges from 20 °C to 25 °C. The annual rainfall ranges from 500 to 900 mm (person. comm). Addis Zemen Hospital serves as a referral hospital for 15 health centers. The natural vegetation of this district is almost similar to the Metema district. However, there were no small- and large-scale farms like Metema, and hence, all the lands have been owned by private farms and the two study areas are 225 km apart from each other as depicted on the map below (Figure 1).

### 2.2. Data Collection Methods and Laboratory Diagnosis

Four-year and seven-month data were obtained from Metema and Addis Zemen Hospitals' registration books. During this time, for those VL-suspected cases, who had complete data on sex, age, occupation, residence, month, year, and rK39, test results were included and collected using a questionnaire. While for those suspected cases, if one of the above variables was not registered, then they were excluded.

According to the guideline for the diagnosis, treatment, and prevention of leishmaniasis in Ethiopia [25], patients who have met the VL clinical case definition (fever for more than two weeks, splenohepatomegaly, malaria, and previous VL ruled out) should be tested by rK39 rapid diagnostic test (RDT) and RDT-positive patients are advised to VL treatment. At both health facilities, the type of rK39 RDT used was DiaMed-ITLEISH-Bio-Rad Laboratories (Marnes-la-Coquette, France). The test was performed according to the manufacturer's instructions.

### 2.3. Data Analysis

After collection, data were entered into an Excel sheet and transferred to Statistical Package for Social Sciences (SPSS) version 20. Line graphs were used to show the monthly and yearly trends of VL, whereas bar graph was used to show the occupational status. A chi-square test was used to see whether there was any association between demographic variables with VL. Finally, p < 0.05 was considered as statistically significant.

## 3. Results

Of the 2,703 VL-suspected cases who were diagnosed with the rK39 test at the two health facilities from October 2017 to May 2021, 877 (32.4%) were confirmed (positive) cases. Of the 2,703 suspected cases, 2,216 (81.9%) and 487 (18.1%) were men and women, respectively. In this study, the majority of 772 (34.8%) of the VL-confirmed (positive) cases were men and there was a significant difference between men and women for VL (*χ*^2^ = 32; p-value < 0.001) (Table 1).

This study revealed that in most of the VL-confirmed (positive) cases, 759 (33.8%) were in age-groups from 15 to 44 years and there were statistically significant differences for VL among age-groups (*χ*2 = 14.9; *p*-value < 0.001). Finally, 526 (33, 4%) farmers were more affected followed by 269 (31,1%) daily laborers (Table 1).

**Figure 2:**
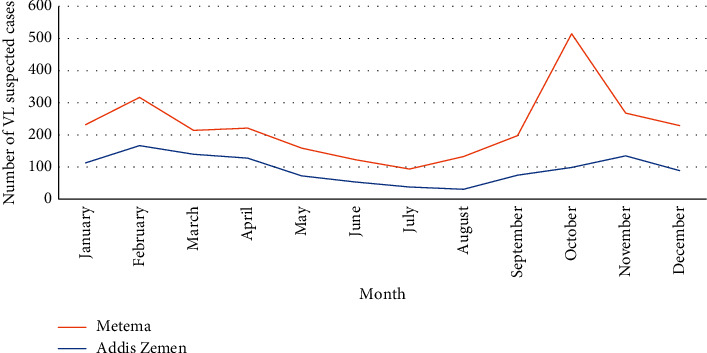
Monthly trends of VL-suspected cases at two health facilities in Ahmara Region northwest Ethiopia from October 2017 to May 2021.

Monthly trends of VL-suspected cases by health facility revealed that more suspected cases were reported from each month throughout the year at Metema Hospital. The largest number of suspected cases was reported in October followed by February. In addition, a dramatic spike was observed from September to October. While decrement in monthly suspected cases was reported from October to November at Metema Hospital (Figure 2).

At Addis Zemen Hospital, the monthly trends depicted that the largest number of suspected cases was reported in November followed by February. In contrast to Metema Hospital's report, there was an increment in suspected cases from August to November (Figure 2).

**Figure 3:**
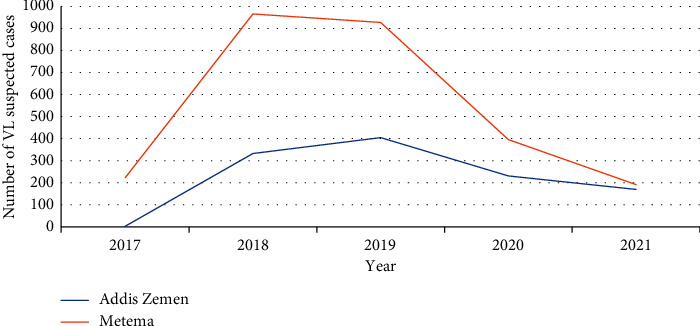
A 4-year and 7-month trend of VL-suspected cases at two health facilities in Ahmara Region northwest Ethiopia from October 2017 to May 2021.

Yearly trends of VL-suspected cases by health facility depicted that more suspected cases were reported from Metema Hospital for the last four-year and seven-month period. Trends of suspected cases at Metema Hospital showed a dramatic increment in the number of suspected cases from 2017 to 2018. The spike-suspected cases were reported in 2018 followed by 2019. Finally, from 2019 to 2020, there was a dramatic decrement in the number of suspected cases (Figure 3).

On the other hand, at Addis Zemen Hospital, there was a gradual increment in the number of suspected cases in contrast to Metema Hospital from 2017 to 2018. From 2018 to 2019, yet there was a slight increment in the number of suspected cases is contrary to the Metema Hospital report. The spike-suspected cases were reported in 2019 followed by 2018 at Addis Zemen Hospital (Figure 3).

The occupation of the VL-suspected cases by health facility depicted that the majority of the suspected cases reported were daily laborers followed by farmers at Metema Hospital, while most of the suspected cases reported at Addis Zemen Hospital were farmers followed by daily laborers (data not shown).

## 4. Discussion

Despite the prevention and control efforts performed in Ethiopia, there are a few reports on VL prevalence and trends at the health facility level. This study revealed that rK39 confirmed VL cases over a four-year and seven-month period to be 32.4%. This finding was higher than the study performed at Abdurafi Health Centre, 21%, Armachiho district [26], and Metema Hospital, 22.6%, Ethiopia [22]. This difference might be due to a greater number of health facilities involved in our study compared to one health facility in other studies.

On the other hand, our finding was lower than 39.1% reported from Addis Zemen Health Centre. This difference might be due to the occurrence of the outbreak from 2005 to 2006 in the Libokemkem district, which might have increased the prevalence of VL [21, 24, 27]. This difference might also be due to differences in laboratory diagnostic methods. In our case, rK39 was used as opposed to the direct agglutination test (DAT), which is more sensitive than rK39 [28], which was used previously in Addis Zemen Health Centre.

The largest number of rK39 VL-confirmed cases 33.8% was found in age-groups of 15-44 years. This was in line with a previous report from Metema Hospital, in which 15-29 years old study subjects accounted for 24.2% [22] But, our finding was different from previously reported from Addis Zemen Health Centre [21]. This might be due to the age classification difference between our and their studies and an outbreak of VL in the Libokemkem district back in 2005 and 2006 [21, 24, 27].

Men were more affected with VL as compared to women (43.8% vs 21.5%). This result was in line with previous findings separately reported from Addis Zemen Health Centre (40.1% vs 36.5%) [21] and Metema Hospital (23.2% vs 18.8%) [22]. The difference in the prevalence of VL between the two sexes might be due to the habit of men sleeping outdoor at night and exposing themselves to sand fly biting [21, 24, 27].

Finally, farmers were the most affected people accounting for 33.4% of the rK39 VL-confirmed cases. This might be due to their daily outdoor activities exposing themselves to sand fly vectors [24, 27].

Monthly trends of VL suspected cases showed that more cases were found in Metema Hospital than in Addis Zemen every month of the year throughout the study period (Figure 2). This might be due to the migration of daily laborers to Metema [20, 29] and due to intense transmission because of differences in altitude, temperature, and rainfall between the two locations.

The largest numbers of suspected cases were reported in October in our study (Figure 2). This result agreed with Humera and Metema Hospitals [22, 29]. But, this result was different from this study at Addis Zemen Hospital (the spike number was in November) (Figure 2). This might be due to the early rainfall stoppage in the Metema district compared to Addis Zemen (Libokemkem district). This created a favorable condition for the early breeding of sand fly vectors and increased vector density after the rainy season [30].

In this study, at Addis Zemen Hospital, there was an increment in the number of suspected cases from August to November unlike at Metema Hospital (Figure 2). This result was in line with the study performed at Metema and Humera Hospitals [22, 30]. However, it was different from this study at Metema Hospital report (Figure 2). This difference might be due to the difference in altitude, temperature, and rainfall for sand fly breeding and infection for humans (Addis Zemen vs Metema areas).

Generally, the overall monthly trends of suspected cases from two health facilities depicted undulating patterns and gave different information with a greater number of suspected cases at Metema Hospital throughout the study period (Figure 2). This might be due to differences in altitude, temperature, and rainfall for favorable conditions to sand fly vectors and intense transmission in the Metema district.

Yearly trends of VL-suspected cases by health facilities depicted that more suspected cases were found from Metema Hospital (1561 vs 1142) (Figure 3). This might be due to many migrant workers (daily laborers) coming from the nonendemic area especially from June to December for weeding and harvesting purposes [20], and this situation might have increased the report throughout the study period. Our trend analysis of VL at Metema Hospital showed a dramatic increment in VL-suspected cases between the years 2017 and 2018, and the spike was observed in 2018 (Figure 3). This is because of the occurrence of the outbreak from 2017 to 2018 (person. comm).

Even if the overall yearly trends of VL-suspected cases at Addis Zemen Hospital look similar to the Metema Hospital, the peak-suspected cases were reported in 2019 (Figure 3). The increment in 2019 at Addis Zemen Hospital was because of the daily laborers being infected while they were at Metema district in 2018 (20, person. comm) and returned to their birthplaces in and around Addis Zemen and diagnosed at Addis Zemen Hospital.

The overall VL-suspected trend showed fluctuating pattern and generally seemed constant over time from two health facilities (Figure 3). This result was in line with the study performed at Addis Zemen Health Centre [21] but different from Metema and Humera Hospitals [22, 29]. This variation might be due to the prevention and control strategies set by the Ethiopian Ministry of Health [25].

In this study, the majority of the suspected cases reported from Metema Hospital were daily laborers (data not shown). This result was in line with the study performed at Metema and Humera Hospitals [21, 29]. But, this is different from our study at Addis Zemen Hospital. This was because of the migration of daily laborers to the Metema district from June to December [20, 29].

### 4.1. Limitations of the Study

This study was carried out in a health institution, and the result may not infer from the general population. In addition, this study depends on serological tests only and false-positive and/or false-negative results might be reported. To fill these gaps, there is a need for further study at the community level using different study designs and more sensitive laboratory diagnostic methods.

## 5. Conclusions

In this study, the total rK39-confirmed VL cases at two health facilities were 877(32.4%). The overall monthly trends of suspected cases from two health facilities depicted undulating patterns and gave different information with a greater number of suspected cases at Metema Hospital throughout the study period. In addition, yearly trends of VL-suspected cases showed fluctuating patterns and generally seemed constant over time from two health facilities. Finally, the majority of the suspected cases reported from Metema Hospital were daily laborers. Because of these daily laborers' yearly migration, what is new in this study was that the Regional Health Bureau should design intervention methods not only in the endemic areas but also in the nonendemic areas where the daily laborers came from.

## Figures and Tables

**Table 1:** Sociodemographic characteristics and rK39 results of VL suspected cases at two health facilities in Amhara Region northwest Ethiopia from Oct 2017 to May 2021.

rk39 results					
Characteristic	Positive N (%)	Negative N (%)	Total N (%)	Χ2	p value
Sex	772 (43.8)	1444 (65.2)	2216 (81.9)	23	0.001
Female	105 (21.5)	382 (78.4)	487 (18.1)		
Total	877 (32.4)	1826 (67.6)	2703 (100)		
Age-group					
< 5	11 (21.2)	41 (78.8)	52 (1.9)	14.9	0.005
5-14	39 (26.4)	109 (73.6)	148 (5.5)		
15-44	759 (33.8)	1481 (66.1)	2240 (82.8)		
≥ 45	68 (25.8)	195 (74.1)	263 (9.7)		
Total	877 (32.4)	1826 (67.6)	2703 (100)		
Residence					
Urban	50 (38.5)	80 (61.5)	130 (4.8)	23	0.133
Rural	827 (32.1)	1746 (67.9)	2573 (95.2)		
Total	877 (32.4)	1826 (67.6)	2702 (100)		
Occupation					
Daily laborer	269 (31.1)	596 (68.9)	865 (32)	1.6	0.443
Farmer	526 (33.4)	1048 (66.9)	1574 (58.2)		
Student	82 (31.1)	182 (68.9)	264 (9.8)		
Total	877 (32.4)	1826 (67.6)	2703 (100)		

## Data Availability

All data are included within the manuscript.

## Abbreviations

VL: Visceral leishmaniasis
CL: Cutaneous leishmaniasis
rK39: A 39-amino-acid-repeat recombinant leishmanial antigen from *Leishmania chagasi*
APHI: Amhara Public Health Institute
DAT: Direct agglutination test.
